# Lack of NLRP3-inflammasome leads to gut-liver axis derangement, gut dysbiosis and a worsened phenotype in a mouse model of NAFLD

**DOI:** 10.1038/s41598-017-11744-6

**Published:** 2017-09-22

**Authors:** Irene Pierantonelli, Chiara Rychlicki, Laura Agostinelli, Debora Maria Giordano, Melania Gaggini, Cristina Fraumene, Chiara Saponaro, Valeria Manghina, Loris Sartini, Eleonora Mingarelli, Claudio Pinto, Emma Buzzigoli, Luciano Trozzi, Antonio Giordano, Marco Marzioni, Samuele De Minicis, Sergio Uzzau, Saverio Cinti, Amalia Gastaldelli, Gianluca Svegliati-Baroni

**Affiliations:** 1 Department of Gastroenterology, Università Politecnica delle Marche, Ancona, Italy; 2 Cardiometabolic Risk Lab, Institute of Clinical Physiology, National Council of Research (CNR), Pisa, Italy; 3 Porto Conte Ricerche, Parco Scientifico e Tecnologico della Sardegna, Alghero, Italy; 4 Department of Biomedical Sciences, Università di Sassari, Sassari, Italy; 5 Department of Experimental and Clinical Medicine, Università Politecnica delle Marche, Ancona, Italy; 6 Obesity Center, Università Politecnica delle Marche, Ancona, Italy

## Abstract

Non-Alcoholic Fatty Liver Disease (NAFLD) represents the most common form of chronic liver injury and can progress to cirrhosis and hepatocellular carcinoma. A “multi-hit” theory, involving high fat diet and signals from the gut-liver axis, has been hypothesized. The role of the NLRP3-inflammasome, which senses dangerous signals, is controversial. Nlrp3^−/−^ and wild-type mice were fed a Western-lifestyle diet with fructose in drinking water (HFHC) or a chow diet. Nlrp3^−/−^-HFHC showed higher hepatic expression of PPAR γ2 (that regulates lipid uptake and storage) and triglyceride content, histological score of liver injury and greater adipose tissue inflammation. In Nlrp3^−/−^-HFHC, dysregulation of gut immune response with impaired antimicrobial peptides expression, increased intestinal permeability and the occurrence of a dysbiotic microbiota led to bacterial translocation, associated with higher hepatic expression of TLR4 (an LPS receptor) and TLR9 (a receptor for double-stranded bacterial DNA). After antibiotic treatment, gram-negative species and bacterial translocation were reduced, and adverse effects restored both in liver and adipose tissue. In conclusion, the combination of a Western-lifestyle diet with innate immune dysfunction leads to NAFLD progression, mediated at least in part by dysbiosis and bacterial translocation, thus identifying new specific targets for NAFLD therapy.

## Introduction

Non-Alcoholic Fatty Liver Disease (NAFLD) is the most common form of chronic liver disease, with prevalence estimates ranging from 25–45% of the adult population and increasing in parallel with that of obesity and diabetes in Western world^1^. NAFLD was first described in 1980 and is divided into the histological categories of (1) Non-Alcoholic Fatty Liver, which includes patients with isolated hepatic steatosis and patients with steatosis and mild non-specific inflammation, and (2) Non-Alcoholic Steatohepatitis (NASH), which is distinguished from the former by the additional presence of features of hepatocellular injury with or without fibrosis^2,3^. Among patients with NAFLD, those with NASH are much more likely to progress to cirrhosis and hepatocellular carcinoma (HCC) than those with only hepatic steatosis, and these conditions are predicted to become the most common indication for liver transplantation^1,4^. NAFLD is associated with features of modern lifestyle, characterized by increased dietary caloric intake of saturated and *trans-*unsaturated fatty acids (FAs), sugar-sweetened beverages and sedentary lifestyle^5–7^. A significant association has been found between fructose intake and the prevalence of diabetes, obesity and NAFLD and the degree of fibrosis in NASH^8^.

In recent years, a key role of the gut microbiota in the pathogenesis of obesity and NAFLD has been identified. Preclinical studies have shown that transplantation of microbiota from obese to lean mice was associated with the occurrence of metabolic alterations in the recipients^9^. Many interactions of gut microbiota with food, bile components and intestinal epithelium have been demonstrated to contribute to NAFLD pathogenesis and progression. The predominant mechanisms are an increased energy harvesting and alterations of intestinal barrier function, which can lead to translocation of bacterial products into the portal circulation and activation of inflammatory processes^9,10^. High fat diet (HFD) has been demonstrated to strongly affect gut microbiota composition, by increasing the abundance of energy harvesting microorganisms, such as Firmicutes and Proteobacteria and by decreasing Bacteroidetes^11–14^. On this regard, inflammasomes are multiprotein complexes that orchestrate host defense mechanisms against infectious agents but their contribution to innate immunity might likely include the control of gut microbiota load and composition^15^. In agreement with this, genetic NLRP3-inflammasome deficiency-associated dysbiosis resulted in abnormal accumulation of bacterial products into the portal circulation and increased severity of liver injury during a methionine/choline-deficient diet (MCD)^16^. Moreover, NLRP3-inflammasome deficient mice develop exacerbated colitis in the dextran sulfate sodium (DSS) model^17–19^.

NLRP3 is also implicated in the pathogenesis of cardiovascular disease, obesity and type-2-diabetes, while controversial data exist on inflammasome activation in liver disease^20–23^. We have previously reported that during the development of liver injury, inflammasome components were upregulated in the liver and downregulated in the gut and this was associated with microbiota modifications and bacterial translocation^24^. On the other hand, an NLRP3 selective inhibitor improved NAFLD pathology and fibrosis in obese diabetic mice^25–27^. Thus, a complex balance exists between diet, gut microbiota, intestinal homeostasis and NLRP3 function, and controversial results have been provided concerning their respective role in the progression of liver injury. Thus, aim of this study was to provide an in depth evaluation of the relationship between innate immunity and Western-lifestyle diet in the progression of NAFLD.

## Results

### Anthropometric parameters and hepatic lipid accumulation

**Figure 1:**
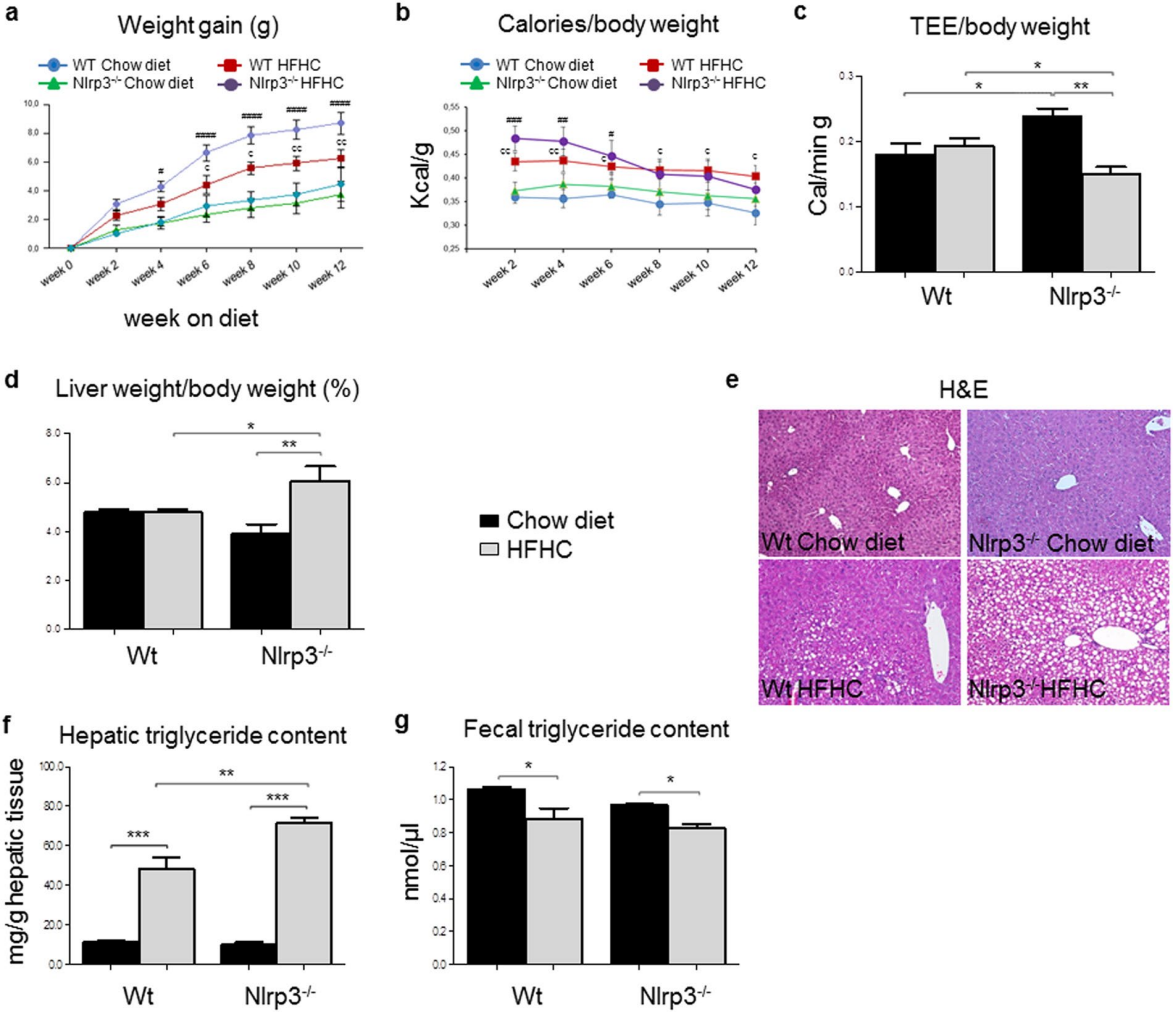
Nlrp3^−/−^-HFHC mice showed increased weight gain and hepatic steatosis. Nlrp3^−/−^-HFHC mice showed increased weight gain (**a**), despite reduced calories intake/body weight ratio (**b**) and this was associated with reduced total energy expenditure (TEE) (**c**). Liver-to-body-weight ratio (**d**) correlated with hepatic lipid accumulation, evaluated by H&E staining (**e**) and triglyceride quantification in liver (**f**). These effects did not correlate with different intestinal lipid absorption (**g**). Mean ± SE: ^#^p < 0.05 vs Nlrp3^−/–^-Chow diet; ^##^p < 0.01 vs Nlrp3^−/−^-Chow diet; ^###^p < 0.001 vs Nlrp3^−/−^-Chow diet; ^####^p < 0.0001 vs Nlrp3^−/−^-Chow diet; ç p < 0.05 vs Wt-Chow diet; çç p < 0.01 vs Wt-Chow diet; *p < 0.05; **p < 0.01; ***p < 0.001.

Nlrp3^−/−^-HFHC gained more weight compared to WT-HFHC mice (Fig. 1a) despite a progressive reduction in the caloric intake/body weight ratio (Fig. 1b). The increased body weight in Nlrp3^−/−^-HFHC was associated with reduced total energy expenditure (TEE) (Fig. 1c). This higher average body weight correlated with an increased liver-to-body weight ratio in Nlrp3^−/−^-HFHC-fed mice (Fig. 1d). To assess whether the increased liver weight observed in Nlrp3^−/−^-HFHC-fed mice was associated with higher fat deposition in the liver, we evaluated hepatic fat content. First, liver sections were stained for H*ematoxylin and Eosin (*H&E). H&E staining revealed features of micro and macrovesicular steatosis in HFHC-fed mice, which were higher in Nlrp3^−/−^ mice (Fig. 1e). To further confirm the increased lipid deposition in the liver of mice lacking NLRP3, we quantified triglyceride content, which was found significantly enhanced in these mice compared to WT animals (p < 0.01) (Fig. 1f). Lastly, to study whether the changes in body weight and composition could reflect different intestinal lipid absorption, quantification of triglycerides in the stool was performed. This analysis revealed that HFHC diet decreased faecal excretion of triglycerides compared to chow diet independently from the genotype, thus indicating that differences in lipid absorption cannot explain the higher hepatic triglyceride content in Nlrp3^−/−^-HFHC mice. (Fig. 1g).

### Effect of HFHC and genetic background on adipose tissue inflammation

**Figure 2:**
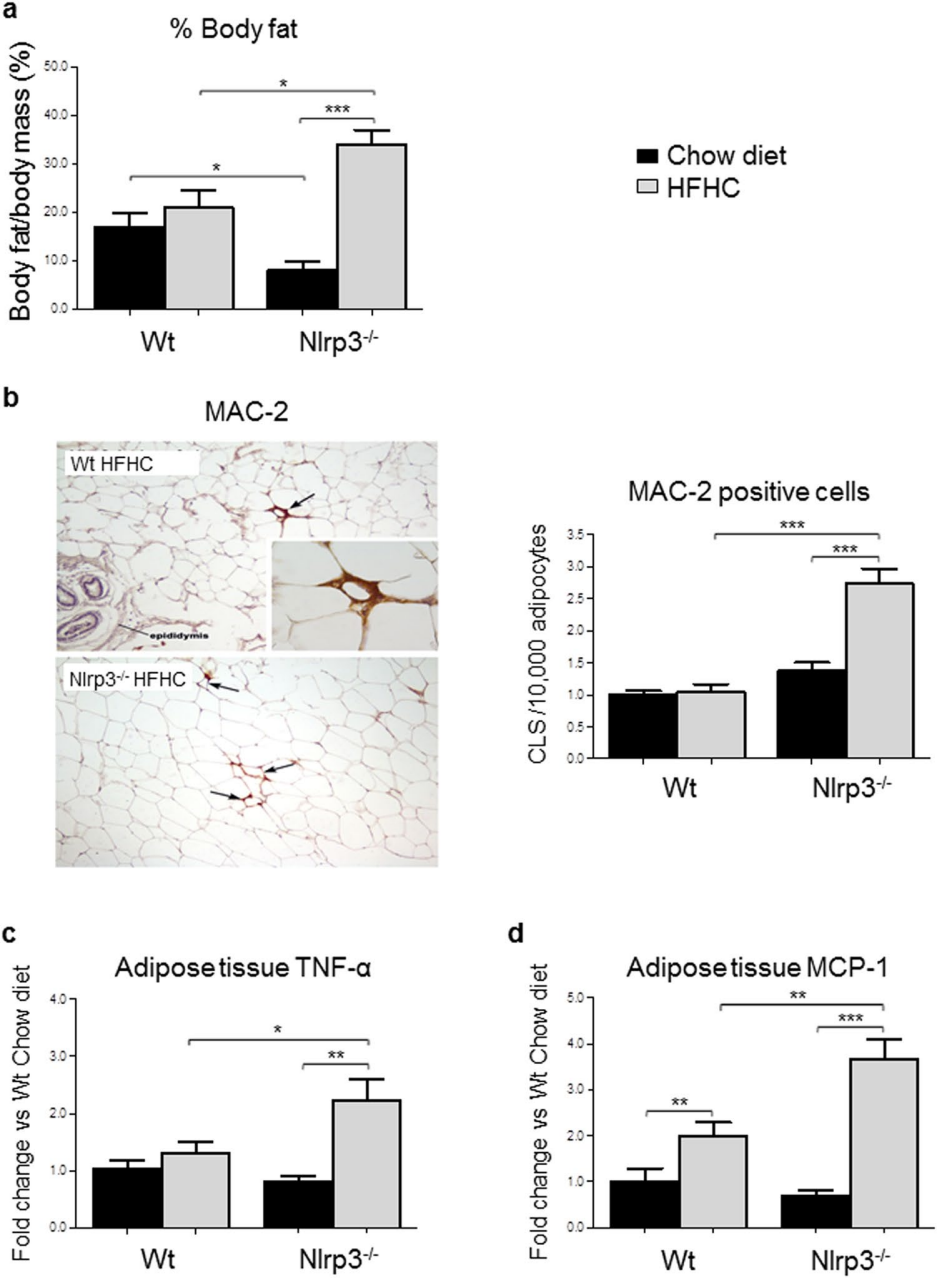
Adipose tissue inflammation was higher in Nlrp3^−/−^-HFHC mice. Percentage of fat mass was increased in Nlrp3^−/−^-HFHC (**a**). MAC-2 immunohistochemistry (magnification, 40×) showed adipose tissue inflammation in Nlrp3^−/−^-HFHC mice (**b**). Gene expression of adipose tissue TNF-α (**c**) and MCP-1 (**d**) evaluated by qRT-PCR was also increased in Nlrp3^−/−^-HFHC mice. Mean ± SE: *p < 0.05; **p < 0.01; ***p < 0.001.

Increased adiposity and adipocyte dysfunction are known to contribute to metabolic diseases by altering adipose tissue-derived secretory factors, and adipose tissue inflammation represents a main mechanism in the pathogenesis of NAFLD^5–7^. Nlrp3^−/−^-HFHC mice had a greater increase in body fat mass (Fig. 2a) that was associated with adipose tissue inflammation. To assess the degree of fat inflammation, a morphological analysis by immunostaining of epididymal fat with the anti-MAC-2 antibody was performed. MAC-2 is a protein known to be expressed by activated macrophages that infiltrate hypertrophic, obese fat and surround death adipocytes, giving rise to distinctive morphological pictures called crown-like structures (CLS)^28^. Nlrp3^−/−^-HFHC mice exhibited a significant increase of the density of CLS when compared with WT-HFHC (Fig. 2b). Similarly, mRNA expression of tumor necrosis factor-α (TNF-α) and monocyte chemoattractant protein-1 (MCP-1) in adipose tissue was significantly increased in Nlrp3^−/−^-HFHC mice (Fig. 2c,d). Thus, the combination of immunohistochemical and gene expression data indicates a higher degree of adipose tissue inflammation in Nlrp3^−/−^-HFHC mice.

### Alterations in hepatic lipid metabolism

**Figure 3:**
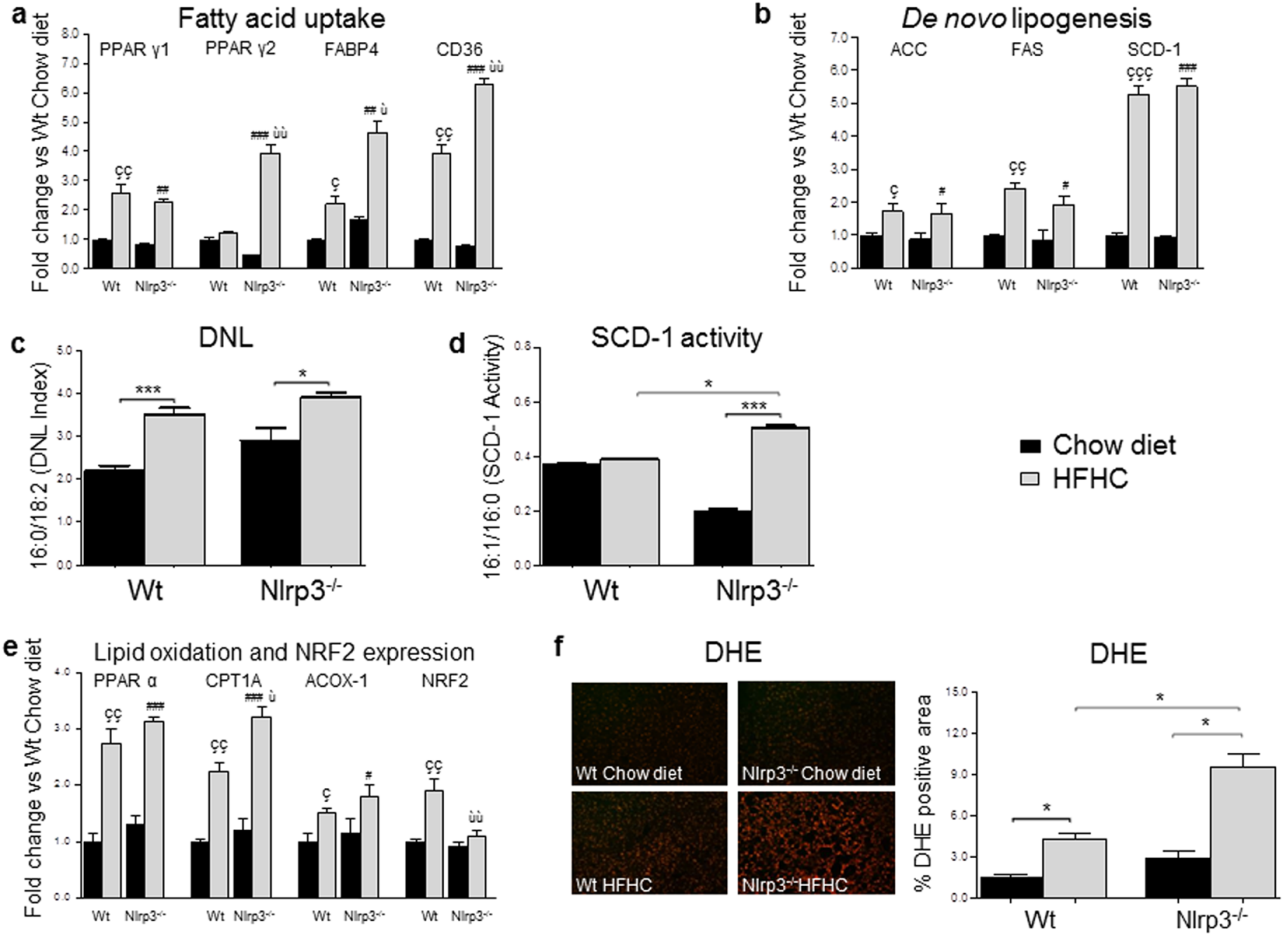
Nlrp3^−/−^-HFHC showed higher hepatic lipid uptake and increased ROS production. HFHC increased PPAR γ1 in a genotype-independent manner, whereas Nlrp3^−/−^-HFHC mice showed higher expression of PPAR γ2 and its downstream effectors FABP4 and CD36 (**a**). Gene expression of ACC, FAS and SCD-1 (**b**). Measurements of plasma indexes of *de novo* lipogenesis and SCD-1 activity (**c**–**d**). Gene expression of PPAR α and of its downstream genes CPT1A and ACOX-1 and expression of the regulator of antioxidant response NRF2 (**e**). Representative images of liver sections stained with DHE (magnification, 20×) and its morphometric analysis (**f**). Mean ± SE: çp < 0.05 vs Wt-Chow diet; ççp < 0.01 vs Wt-Chow diet; çççp < 0.001 vs Wt-Chow diet; ^#^p < 0.05 vs Nlrp3^−/−^-Chow diet; ^##^p < 0.01 vs Nlrp3^−/−^-Chow diet; ^###^p < 0.001 vs Nlrp3^−/–^Chow diet; ùp < 0.05 vs Wt-HFHC; ùùp < 0.01 vs Wt-HFHC; *p < 0.05; ***p < 0.001.

In addition to adipose tissue evaluation, we measured the expression of genes involved in hepatic lipid metabolism, in order to investigate the mechanisms behind the increased hepatic steatosis. HFHC increased PPAR γ1 independently from the genotype, whereas Nlrp3^−/−^-HFHC mice showed higher gene expression of PPAR γ2 and of its downstream effectors, such as the fatty acid binding protein-4 (FABP4) and CD36, which are involved in lipid uptake and storage (Fig. 3a)^29^. HFHC diet enhanced *de novo* lipogenesis (DNL)^30^, independently from the genotype, as shown by the expression of the acetyl Co-A carboxylase-1 (ACC), fatty acid synthase (FAS) and stearoyl-CoA desaturase-1 (SCD-1) (Fig. 3b). An increased plasmatic ratio of palmitic/linoleic acid (16:0/18:2), as a marker of DNL (Fig. 3c), also confirmed mRNA results. Conversely, palmitoleic/palmitic acid (16:1/16:0) ratio, an index of SCD-1 activity, was increased in Nlrp3^−/−^-HFHC mice only (Fig. 3d). HFHC diet activated hepatic fatty acid oxidation, as shown by the expression of PPAR α and of its downstream genes palmitoyltransferase 1 A (CPT1A, a key enzyme in mitochondrial fatty acids β-oxidation) and Acyl CoA oxidase-1 (ACOX-1), a rate-limiting enzyme in peroxisomal fatty acids β-oxidation) (Fig. 3e)^31^. However, CPT1A expression was significantly increased in Nlrp3^−/−^ compared to WT (p < 0.05), which was associated with lower mRNA levels of the “master regulator” of the antioxidant response NRF2 (Fig. 3e)^32^. Since excessive mitochondrial fatty acid β-oxidation is known to induce oxidative stress^33,34^, we measured the concentration of ROS by dihydroethidium (DHE) staining and found the highest concentration of anion superoxide in Nlrp3^−/−^-HFHC (Fig. 3f). These data indicate that increased steatosis in Nlrp3^−/−^-HFHC might be mediated by higher fatty acid uptake, which activates fatty acid catabolism and oxidative stress, also favored by decreased anti-oxidant response.

### Hepatic injury evaluation

**Figure 4:**
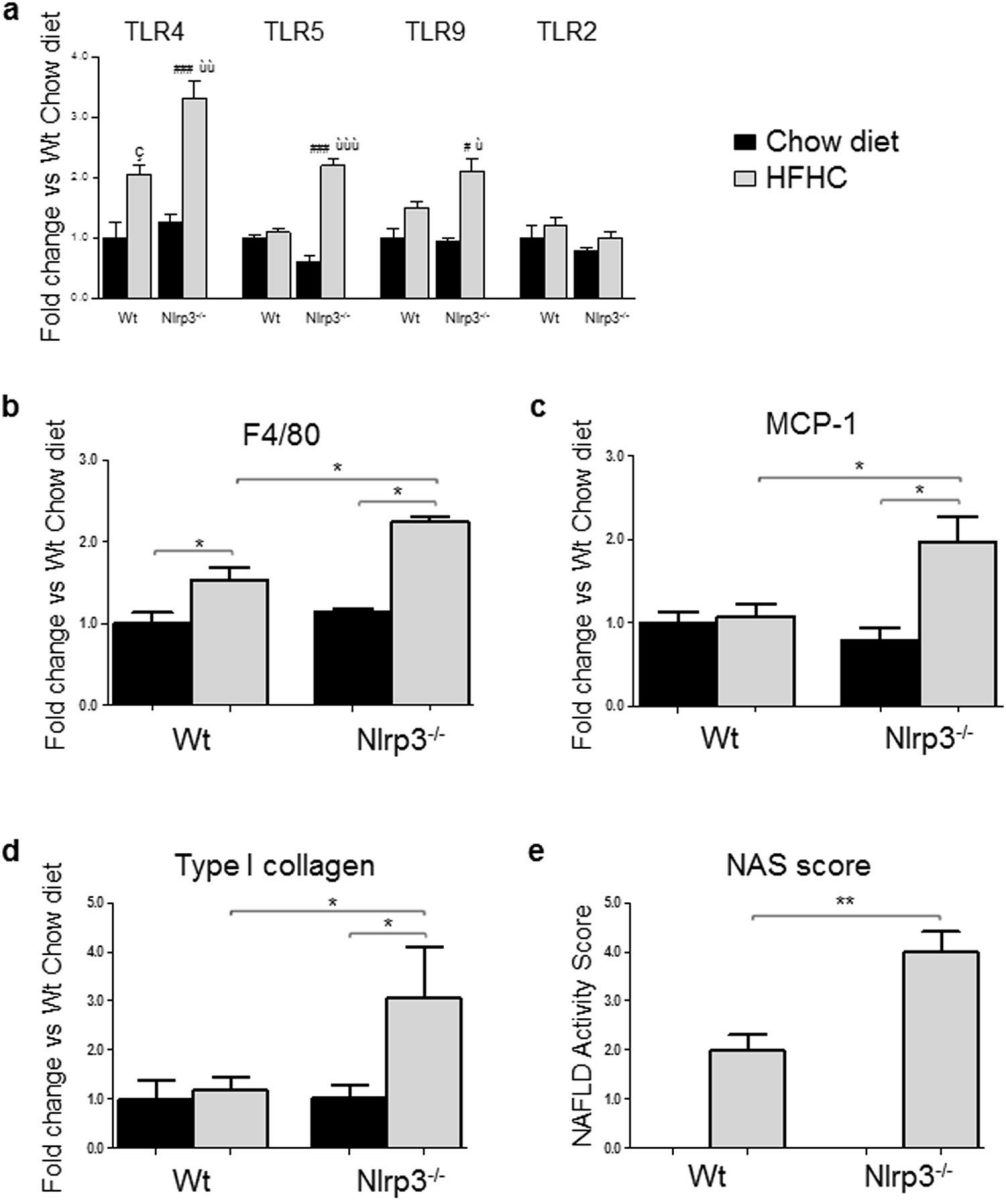
Nlrp3^−/–^HFHC had a more severe liver injury. qRT-PCR showed increased expression of TLR4, TLR5 and TLR9 in Nlrp3^−/−^-HFHC, whereas no differences were observed in TLR2 expression (**a**). F4/80 (**b**), MCP-1 (**c**) and Type I collagen (**d**) gene expression was higher in Nlrp3^−/−^-HFHC. The degree of liver injury was measured according to the Kleiner’s score (**e**). Mean ± SE: çp < 0.05 vs Wt-Chow diet; ^#^p < 0.05 vs Nlrp3^−/−^-Chow diet; ^###^p < 0.001 vs Nlrp3^−/−^-Chow diet; ùp < 0.05 vs Wt-HFHC; ùùp < 0.01 vs Wt-HFHC; ùùùp < 0.001 vs Wt-HFHC; *p < 0.05; **p < 0.01.

Oxidative stress due to ROS accumulation can contribute to either promotion or progression of chronic liver disease. Furthermore, hepatic injury can progress through intracellular pathways mediated by different Toll-like receptors (TLRs) via recruitment of various adaptor proteins^9,10,35^. Nlrp3^−/−^-HFHC mice showed higher expression of TLR4 (that recognizes LPS, a gram-negative bacteria wall component)^36^, TLR5 (known to recognize bacterial flagellin from invading mobile bacteria)^37^ and TLR9 (a specific receptor for double-stranded bacterial DNA)^36^ compared to WT-HFHC (Fig. 4a). No differences were observed in TLR2 expression, that mediates host response to gram-positive bacteria (Fig. 4a)^36^. HFHC diet increased mRNA expression of F4/80, a marker of macrophage infiltration, which was further enhanced in Nlrp3^−/−^ mice (Fig. 4b). Differently, gene expression of the pro-inflammatory cytokine MCP-1 and of Type I collagen were increased only in Nlrp3^−/−^-HFHC-fed mice (Fig. 4c,d). Finally, hystological evaluation assessed by the NAS score^38^ showed the highest degree of liver injury in Nlrp3^−/−^-HFHC-fed mice (Fig. 4e).

### Intestinal permeability and bacterial translocation

**Figure 5:**
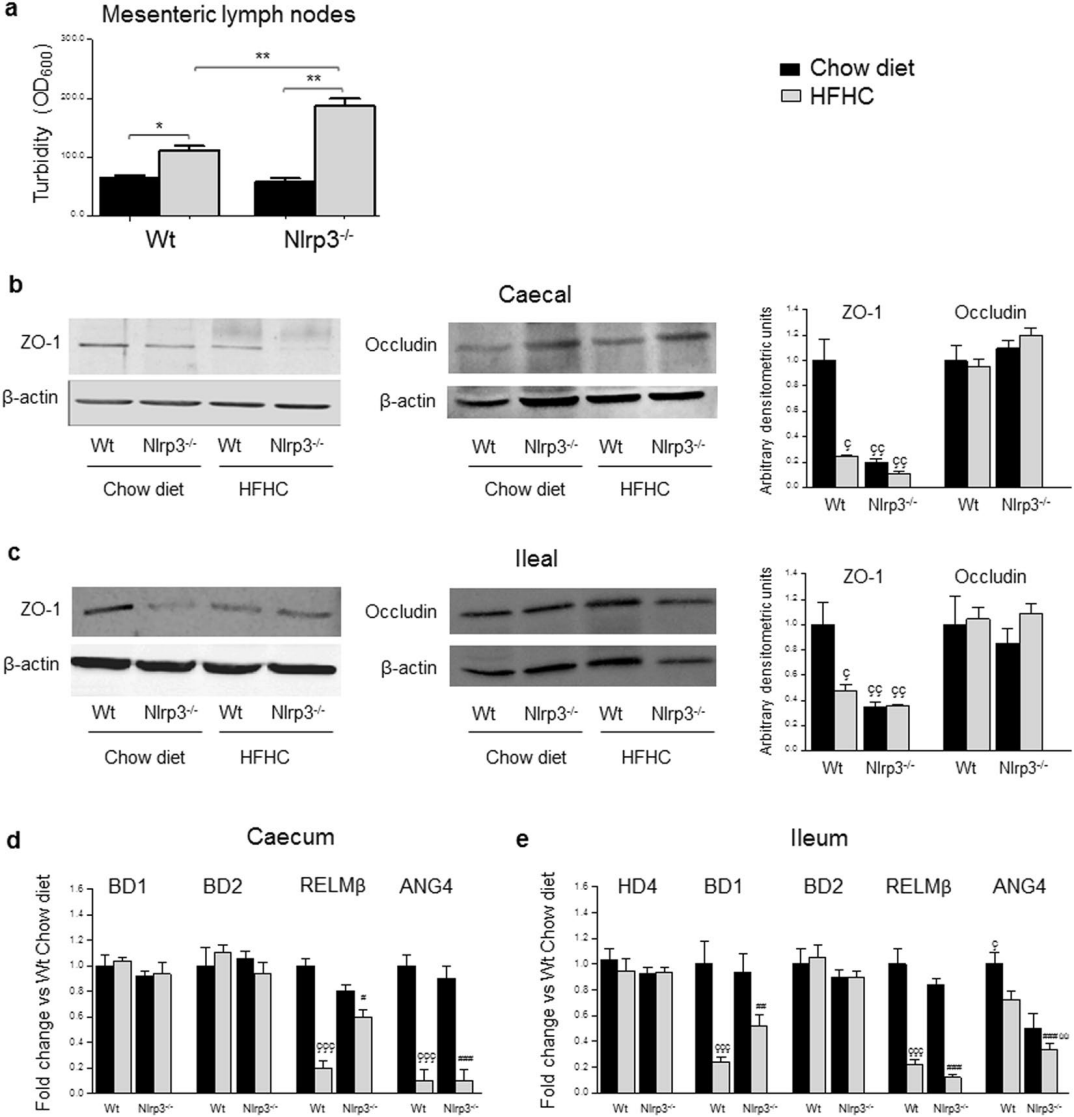
Nlrp3^−/−^-HFHC-fed mice showed increased bacterial translocation and AMPs. HFHC induced bacterial translocation, expressed as turbidity of cultured mesenteric lymph nodes, that was further increased in Nlrp3^−/−^-HFHC-fed mice (**a**). Representative cropped Western blotting and densitometric analysis for caecal and ileal tight junction proteins (**b**-**c**) (full-length blots are presented in Supplementary Fig. S2). Regarding AMPs, in the caecum β-defensin 1 and 2 (BD1–2) expression was unchanged, whereas a lower expression of resistin-like molecule β (RELMβ) and angiogenin 4 (ANG4) has been observed after HFHC diet but independently from the genotype (**d**). In the ileum, HD4 and BD2 gene expression was not modified by neither diet or genotype, BD1 and RELMβ were reduced in HFHC-fed mice, whereas diet-induced reduction of ANG4 was more pronounced in NLRP3-deficient mice (**e**). Mean ± SE: *p < 0.05; **p < 0.01; ç p < 0.05 vs Wt-Chow diet; çç p < 0.01 vs Wt-Chow diet; ççç p < 0.001 vs Wt-Chow diet; ^#^p < 0.05 vs Nlrp3^−/−^-Chow diet; ^##^p < 0.01 vs Nlrp3^−/−^-Chow diet; ^###^p < 0.001 vs Nlrp3^−/−^-Chow diet; ùù p < 0.01 vs Wt-HFHC.

Western diet-induced NAFLD can be associated with dysbiosis, gut barrier dysfunction and increased intestinal permeability leading to bacterial translocation, that drives the progression toward NASH^9^. We then looked at mesenteric lymph nodes cultures, as a measure of bacterial translocation^24^, and found the highest increase in bacterial growth in Nlrp3^−/−^-HFHC (Fig. 5a), indicating a combined role of Western-lifestyle diet and NLRP3 deficiency in inducing bacterial translocation. The maintenance of a proper gut barrier against bacterial translocation is mediated by several mechanisms, including the secretion of enzymes/proteins and mechanical barrier such as the presence of tight junctions, which exert a key role in the protection against intraluminal microorganisms^39^. To study the eventual alterations of intestinal barrier associated to bacterial translocation, Western blot for tight junction proteins was performed both in the caecum and in the ileum. Caecal Zonula Occludens-1 (ZO-1), an intestinal protein which binds different transmembrane proteins and modulates intestinal permeability by disassembling the intercellular tight junctions^40^, was decreased in HFHC-fed mice and in chow-fed Nlrp3^−/−^ (Fig. 5b). Differently, protein expression of caecal Occludin, a transmembrane protein which forms the core of the tight junctions and controls ion selectivity and permeability of the paracellular pathway between adhering cells^41^, did not change (Fig. 5b). To also evaluate eventual alterations of the gut barrier in the upper part of the intestine, we analyzed ZO-1 and Occludin protein expression in the ileum and found a trend similar to what was observed in the caecum (Fig. 5c).

Thus, we measured the expression of antimicrobial peptides (AMPs) both in large and small intestine (Fig. 5d,e). At colon level, expression of β-defensin 1 and 2 (BD1-2), antimicrobial peptides produced by epithelial cells, did not change among the experimental groups (Fig. 5d)^39^. The expression of the inducible BD2 was also not modified in the ileum, where we could observe a diet-dependent decrease of the constitutive BD1 (Fig. 5e). α-defensin 4 (HD4) expression, the most abundant peptide produced by ileal Paneth cells, was unaffected by both diet and genetic background (Fig. 5e). A lower expression of Resistin-like molecule β (RELMβ), an antimicrobial peptide secreted by goblet cells, which stabilizes mucin polymer and regulates intestinal mucin secretion^42^, was found decreased in both WT and Nlrp3^−/−^ mice after HFHC, either in the caecum or in the ileum (Fig. 5d,e). Finally, Angiogenin-4 (ANG4), an antimicrobial peptide which belongs to a family of RNases and possesses a well-known antibacterial and antiviral function^42^, was significantly lower in HFHC-fed mice, independently from the genotype in the caecum, whereas was significantly (p < 0.01) reduced in the ileum of Nlrp3^−/−^-HFHC mice compared to WT-HFHC (Fig. 5d,e). Taken together these results indicate that HFHC alters the physical and chemical mechanisms involved in the maintenance of gut barrier functions, by impairing tight junctions expression and AMPs secretion. In addition, NLRP3 deficiency further affects intestinal barrier by decreasing the expression of ANG4. Thus, our data suggest that the combination of a Western-lifestyle diet with immunological dysregulation due to NLRP3 deficiency alters intestinal homeostasis leading to increased bacterial translocation.

### Dietary and genetic background effects on microbiota community

The Western-lifestyle diet can induce gut dysbiosis that, in turn, has been implicated in bacterial translocation and worsening of liver injury in NAFLD^9^. Further, lack of NLRP3 affects, *per se*, the composition of gut microbiota^16^. However, no data exist regarding the effect of a Western-lifestyle diet on Nlrp3^−/−^ host gut microbiota. Thus, we evaluated the different gut microbial composition that possibly occurs in WT and Nrlp3^−/−^ mice with either HFHC or chow diet (Supplementary Fig. S1). The microbial communities associated to each group were compared according to their α-diversity, richness and β-diversity. A dramatic reduction in α-diversity and richness values was induced by HFHC independently from the genotype. Analysis of β-diversity clearly illustrates group-level differences in the taxonomic composition, with the highest variation according to diet treatment and the variation due to genetic background being more evident in HFHC-fed mice (Supplementary Fig. S1).

**Figure 6:**
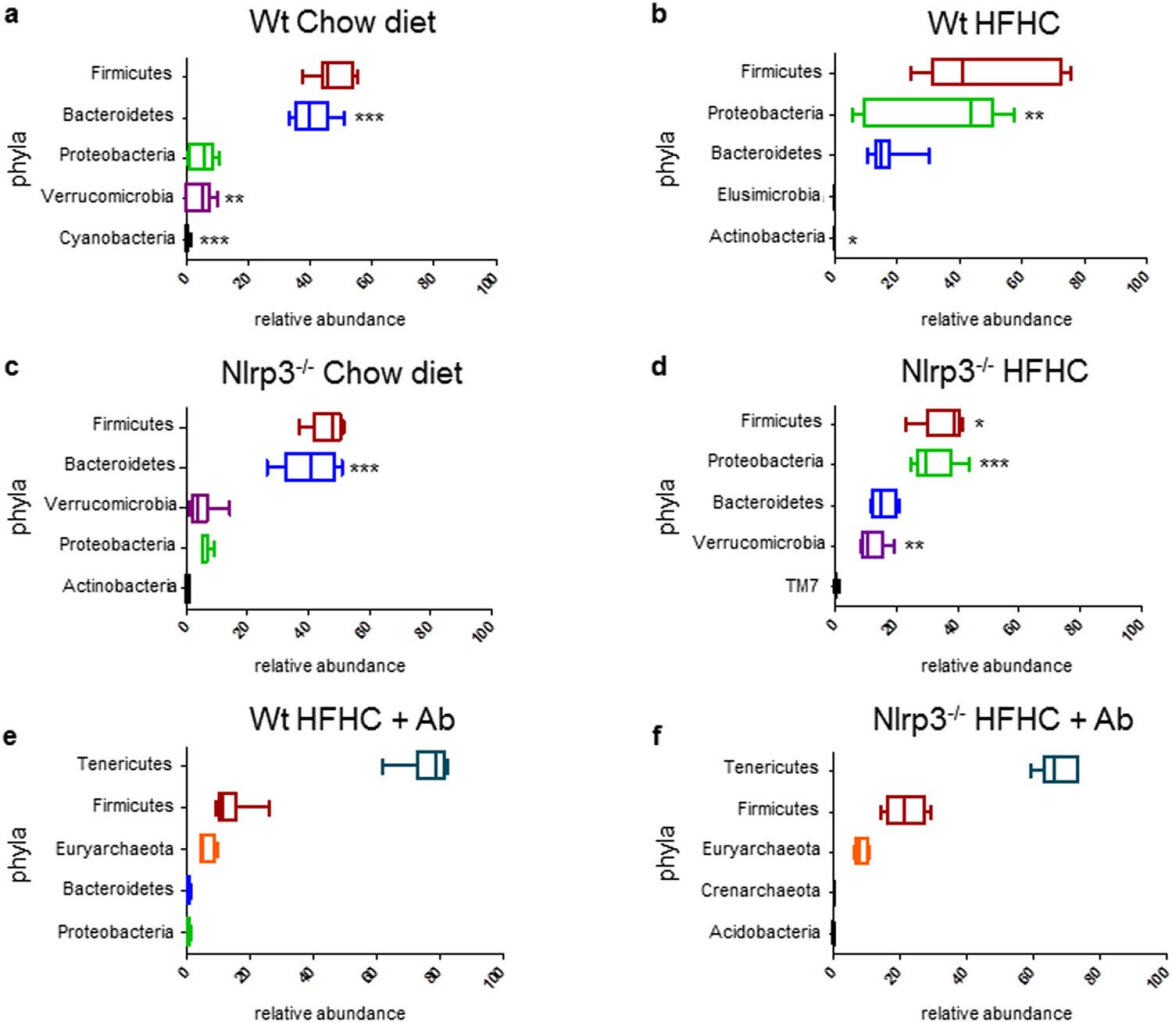
Effect of diet and genotype on gut microbiota composition. Boxplots showing the most abundant microbial phyla. Features are ordered by decreasing median of the relative abundance among subjects. Boxplots are colored based on the relative phylum. Asterisks indicate statistical significance of phylum changes for each genotype when comparing the two diets (*p < 0.05; **p < 0.01; ***p < 0.001).

According to NLRP3-inflammasome deficiency and diet, significant differences between microbial taxa were found (Fig. 6). In WT mice, as expected, Firmicutes/Bacteroidetes ratio was higher in HFHC-fed animals due to a reduction of Bacteroidetes abundance (Fig. 6b). HFHC also promoted an increased abundance of gram-negative Proteobacteria and a reduction on Verrucomicrobia (Fig. 6b). *Akkermansia* was the most prevalent genus of Verrucomicrobia and accounted for 4.6 ± 4.1% of the microbial community in WT-chow (Fig. 6a) and was almost undetectable (0.04 ± 0.05%) in WT-HFHC (Fig. 6b). The increased abundance of Proteobacteria concerned mostly OTUs classified at the family level as *Desulfovibrionaceae*, including *Desulfovibrio* and *Bilophila* pathobiont genera, representing 56.5% and 42.2% of this family group respectively. In Nlrp3^−/−^ mice, HFHC diet induced similar changes in gut microbial composition but to a higher extent (Fig. 6d). Energy harvesting Firmicutes were significantly higher in HFHC diet compared to Nlrp3^−/−^ mice fed chow, and Proteobateria were more consistently increased (Fig. 6; p < 0.001). Noteworthy, Verrucomicrobia showed great variation in its relative abundance when mice were challenged by diet (HFHC) and by innate immunity impairment (Nlrp3^−/−^) (Fig. 6d). In Nlrp3^−/−^mice fed with chow (Fig. 6c), *Akkermansia* abundance was similar to that observed in WT mice on the same diet (Fig. 6a), while its relative abundance showed an impressive increase at 14.62 ± 5.82% solely in the Nlrp3^−/−^mice fed with HFHC diet (Fig. 6d). Taken together, these data suggest that lack of NLRP3 does not lead to a derangement of gut microbial composition in chow-fed mice. However, the impact of HFHC in mice lacking NLRP3-inflammasome functions was more dramatic than in WT mice, suggesting that this pivotal player of the innate immunity might contribute to control dysbiosis induced by Western-lifestyle diet.

### Effect of antibiotic treatment on microbiota composition, adipose tissue inflammation and liver injury

To confirm that intestinal dysbiosis and permeability contribute to NAFLD severity in Nlrp3^−/−^-HFHC, antibiotic treatment was performed^24^. We first analyzed whether antibiotics could modify microbiome composition. Antibiotic treatment, as expected^16,24^, had a dramatic effect on bacterial composition (Fig. 6e–f and Supplementary Fig. S1). Gram-negative bacteria (i.e., Bacteroidetes and Proteobacteria) were substantially depleted by antibiotics, while, not surprisingly, cell wall-free Tenericutes were the most prevalent taxa adapted to the antibiotic pressure. Similarly, Euryarchaeota was among the most abundant phyla represented after antibiotic treatment, as it comprises a large number of genera either cell-wall free or with a pseudo-peptidoglycan wall (i.e., Methanobacteria) (Fig. 6e,f).

**Figure 7:**
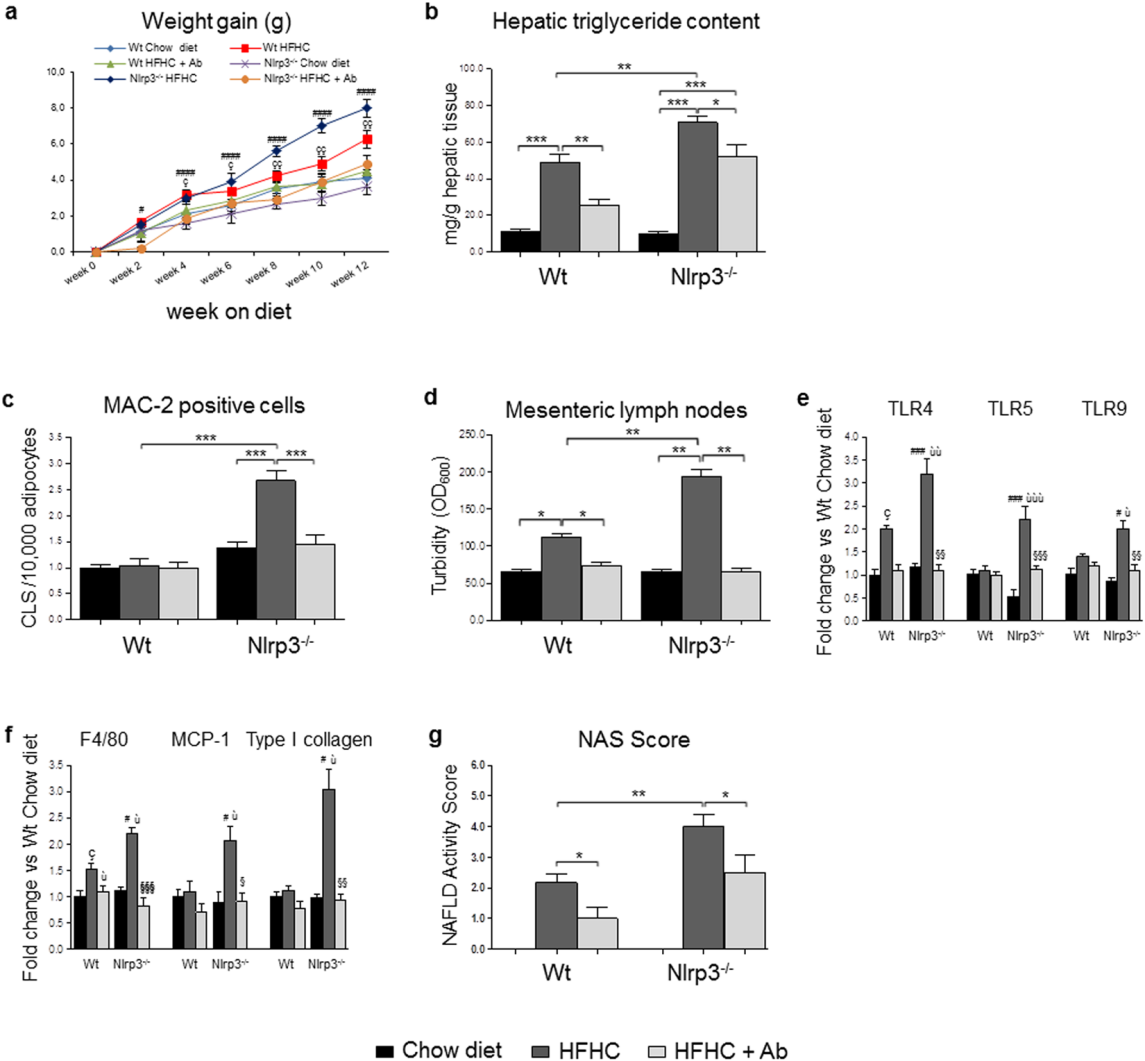
Effect of gut decontamination. Effect of antibiotic treatment on body weight (**a**), hepatic triglyceride deposition (**b**) and adipose tissue inflammation (**c**). Mesenteric lymph nodes colonization was decreased after antibiotics (**d**) and was associated with a significant reduction of hepatic TLR4, TLR5 and TLR9 gene expression (**e**). Antibiotics reduced hepatic gene expression of F4/80, MCP-1 and Type I collagen (**f**) and NAS Score (**g**) in Nlrp3^−/−^-HFHC mice. Mean ± SE: Mean ± SE: *p < 0.05; **p < 0.01; ***p < 0.001; ^#^p < 0.05 vs Nlrp3^−/−^-Chow diet; ^##^p < 0.01 vs Nlrp3^−/−^-Chow diet; ^###^p < 0.001 vs Nlrp3^−/−^-Chow diet; ^####^p < 0.0001 vs Nlrp3^−/−^-Chow diet; çp < 0.05 vs Wt-Chow diet; ççp < 0.01 vs Wt-Chow diet; ùp < 0.05 vs Wt-HFHC; ùùp < 0.01 vs Wt-HFHC; ùùùp < 0.001 vs Wt-HFHC; ^§^p < 0.05 vs Nlrp3^−/−^-HFHC; ^§§^p < 0.01 vs Nlrp3^−/−^-HFHC; ^§§§^p < 0.001 vs Nlrp3^−/–^HFHC.

This modified microbiota was associated with a different phenotype in Nlrp3^−/−^-HFHC mice. Antibiotic treatment was associated with reduced weight gain in Nlrp3^−/−^-HFHC compared to WT-HFHC (Fig. 7a). In Nlrp3^−/−^-HFHC, antibiotics decreased hepatic triglyceride accumulation (Fig. 7b) and adipose tissue inflammation (Fig. 7c). Furthermore, following antibiotic treatment, bacterial growth in cultured lymph nodes was lowered (Fig. 7d) and this was associated with reduced TLR4, TLR5 and TLR9 expression in the liver of Nlrp3^−/−^-HFHC mice (Fig. 7e), indicating a role of bacterial products translocation in these processes. Reduced bacterial translocation in Nlrp3^−/−^-HFHC mice was associated with decreased gene expression of pro-inflammatory markers, such as F4/80 and MCP-1 and of Type I collagen (Fig. 7f) together with a significant reduction in NAS score (Fig. 7g).

## Discussion

An exciting hypothesis in the pathogenesis of NAFLD/NASH is that multiple steps of interaction between intestinal microbiota and the host might be the cause of derangement in either glucose and/or lipid metabolism leading to metabolic diseases^9^. In this scenario, diet-induced intestinal dysbiosis provides pathogen-associated molecular patterns (PAMPs), that cross gut epithelial barrier and activate the innate immune response (by mean of TLRs and “the inflammasomes”) with the release of inducible antimicrobial peptides^16,43–45^. While the lack of inflammasome has been reported to protect from the development of metabolic syndrome and associated diseases^35,46^, this possibility in NAFLD is still debated^16,25^. Activation of inflammasome components has been shown in patients with chronic hepatic injury, particularly NASH, and in experimental models of NAFLD^21,23^. However, lack of NLRP3 was associated with reduced steatosis in some studies but it had no effects in others^21,46–48^. In the choline-deficient L-amino acid-defined (CDAA) model of liver injury, although not associated with features of the metabolic syndrome, inflammasome activation occurred during steatohepatitis development, whereas Nlrp3^−/−^ mice were protected during CDAA treatment^22,23^. More recently, an NLRP3 selective inhibitor improved NAFLD pathology either in the appetite-defective foz/foz mice overnutrition model fed an atherogenic diet or in the MCD model^25^. These controversial results might be explained by the different methods used and by the complex role of inflammasome components in regulating cellular homeostasis^5^.

In the present study, we planned a set of experiments to elucidate the role of inflammasome in the development of NAFLD. Given the pivotal role of food in the development of dysbiosis, metabolic disorders and NAFLD, we used a well-defined model of Western diet associated with increased body weight, fat mass, fasting glucose and insulin-resistance, with development of minimal fibrosis after 12 weeks^49^. In our model, Nlrp3^−/−^ mice showed increased liver steatosis, macrophage infiltration and liver injury (NAS score). Furthermore, Nlrp3^−/−^-HFHC mice increased fat mass and adipose tissue inflammation (MAC-2 positive staining and gene expression of TNF-α and MCP-1 in adipose tissue), indicating the occurrence of “inflamed” adipose tissue and the development of adipose tissue insulin-resistance, that is associated with fatty acids overflow and hepatic fat accumulation as a consequence^50,51^. This study is in agreement with and extends previous observations showing that Nlrp3^−/−^ mice fed MCD diet, a model associated with steatohepatitis but with a cachectic phenotype, had worsened liver injury, and this was attributed to a pathogenetic microbiota^16^. However, no detailed mechanisms on the effect of NLRP3 deficiency were obtained in a Western-lifestyle diet. Studies evaluating the effect of a Western-lifestyle diet on microbiota composition in the presence of innate immunity defects are lacking.

The worsen degree of adipose tissue inflammation and liver injury in NLRP3 deficient mice was associated with specific modifications of gut microbiota composition. Specifically, in the caecal content of Nlrp3^−/−^-HFHC mice we observed: a) an increased abundance of Proteobacteria, the main pathobiont bacteria expressing endotoxins associated with the highest degree of liver injury in a model of HFD and fibrosis^24^, b) a significant increase of mucus degrading bacteria such as *Akkermansia muciniphila* (phylum Verrucomicrobia) and *Desulfovibrio* (phylum Proteobacteria). *Akkermansia muciniphila* is a gut commensal bacteria that resides in the mucus layer and exerts mucin-degrading function with a controversial role in basal metabolism homeostasis and immune tolerance toward commensal bacteria^52^. To this end, preparations of *Akkermansia muciniphila* as therapeutic options to target human obesity and associated disorders have been proposed^53,54^. Indeed, although *Akkermansia* can be considered as a regulator of the thickness of gut barrier, exaggerate mucus degradation has been shown to contribute to intestinal and systemic inflammation by increased layer crossing of luminal antigens^13,55^. More recently, *in vivo* isotope labeling combined with metaproteomics showed that the active microbiome in HFD-fed mice increased bacterial taxa as Verrucomicrobia and *Desulfovibrionaceae*, and this active microbiome affected metabolic pathways such as energy production and carbohydrate metabolism^56^. Again HFD was associated in mice with the expansion of Firmicutes (appearance of Erysipelotrichi), Proteobacteria (*Desulfovibrionales*) and Verrucomicrobia, a decrease in AMPs expression, increased intestinal permeability and finally a decrease in ileal secretion of chloride, likely responsible for massive alteration in mucus phenotype^13^. As suggested in this last study, a collapse of the mucus barrier colonized by a dysbiotic microbiota might promote the emergence of mucus degrading bacteria (*Akkermansia* and *Desulfovibrio*) that may further participate in the alteration of the mucus barrier.

Several mechanisms, in addition to the mucus layer, act simultaneously in order to prevent bacterial translocation, such as the efficiency of the immune function, the production of AMPs, and the presence of active and functional tight junctions^39^. No data are available on the effects of a Western-lifestyle diet on these mechanisms in the presence of innate immunity defects. In our hands, both the Western diet and the genetic background were independently associated with intestinal barrier alterations. While no differences in Occludin were observed, protein expression of ZO-1, which is involved in the assembly of tight junctions^57^ was reduced in WT-HFHC, Nlrp3^−/−^ and Nlrp3^−/−^-HFHC. On the other hand, bacterial translocation was observed in HFHC mice, more prominently in Nlrp3^−/−^, indicating a major role of the modified microbiota on this process.

In our study, no co-housing experiments were performed. Henao-Mejia & coll.^16^ have already shown that in this condition a transmissible microbiota present in inflammasome-deficient mice was the major contributor to NASH progression. In addition, since one of our aims was to study the modifications of the gut microbiota in NLRP3-deficient mice fed a Western-lifestyle diet, we decided not to perform co-housing experiments to avoid any kind of cross contamination that would have provided potentially confusing data without adding any further contribution to the main conclusion of our study.

Concerning the immunological regulation of gut barrier, this results from a complex interplay between microbiota and mucosal response (including AMPs and immunoglobulin secretion), which finely regulate each other. Different studies have demonstrated that lack of inflammasomes can interfere with this delicate equilibrium by dysregulating the secretion of AMPs, such as defensins^43^, and by decreasing goblet cell mucus secretion^58^, which is tightly regulated by RELMβ. On the other hand, a role of NLRP in the regulation of Paneth cells functions (which produce ANG4) has been observed^43^. Furthermore, IL-18, as NLRP3 downstream, has been identified to play a crucial role in the maintenance of intestinal integrity^18^. As shown by Tomas *et al*., decrease in antimicrobial peptide expression was predominantly observed in the ileum of mice after HFD, where bacterial density appeared highest, associated with expansion of *Desulfovibrionales* and Verrucomicrobia^13^. In line with this, our study showed a prominent role of the diet-induced dysbiosis on AMPs secretion and an inflammasome-dependent downregulation of ANG4, an intestinal protein with important antibacterial functions. Thus, a plausible explanation of the observed higher intestinal permeability in Nlrp3^−/−^-HFHC mice is that the altered antimicrobial activity, induced by the combination of a Western-lifestyle diet with NLRP3 deficiency, leads to increased abundance of mucus degrading bacteria, thus favoring the passage of pathogens into the portal circulation.

Bacterial translocation is considered a key mechanism in the pathogenesis of NAFLD/NASH, and growing evidences indicate a role of TLRs in these processes^59^. In our hands, in Nlrp3^−/−^-HFHC, the association of microbiota modifications with intestinal permeability leads to translocation of bacterial products that signal the liver through TLR4 (that recognizes LPS, a gram-negative bacteria wall component)^36^, TLR5 (known to recognize bacterial flagellin from invading mobile bacteria)^37^ and TLR9 (a specific receptor for double-stranded bacterial DNA)^36^ compared to WT-HFHC. No differences were observed in TLR2 expression, that mediates host response to gram-positive bacteria^36^. The key role of gut dysbiosis in the pathogenesis of adipose tissue and hepatic alterations has been proved by antibiotic treatment, which dramatically modified bacterial composition, depleting gram-negative bacteria, including Proteobacteria which strongly correlates with liver disease progression^24^, finally reducing bacterial translocation, liver injury and adipose tissue inflammation. Gut decontamination induced by antibiotics significantly reduced TLRs expression, indicating a major role of gram-negative products translocation in their activation, that was associated with features of NAFLD (increased body weight and hepatic triglyceride deposition) and progression of liver disease (inflammation and fibrogenesis). Our data expand previous observations from Henao-Mejia^16^, showing that colonic dysbiosis is present in mice lacking ASC-inflammasome (a downstream of NLPR3) upon MCD diet, and bacterial products derived from the intestine trigger hepatic TLR4 and TLR9 activation, which was associated with disease progression^16^.

Several metabolic phenomena could explain the higher degree of steatosis and liver injury in Nlrp3^−/−^-HFHC: a) reduced energy expenditure that could explain the increased body weight and fat mass despite a progressive decrease in the caloric intake/body weight ratio in Nlrp3^−/−^-HFHC compared to other groups; b) increased expression of PPAR γ2 and of its downstream effectors, that facilitates fat deposition in the liver in the face of positive energy balance^29,60–62^; c) increased hepatic SCD-1 activity, a central lipogenic enzyme associated with hepatic steatosis^63,64^; d) increased mitochondrial β-oxidation measured by CPT1A expression^31^. No differences between Nlrp3^−/−^ and WT were observed concerning triglyceride content in the stool, fatty acid synthesis and *de novo* lipogenesis. Concerning fatty acid oxidation, increased β-oxidation is a source of ROS and the protective mechanism of PPAR α agonists in NAFLD should thus be associated with enhanced anti-oxidant capacity^31^. However, increased NRF2 expression was found in WT-HFHC as a protective mechanism, whereas no differences compared to controls were observed in Nlrp3^−/−^-HFHC. NRF2 is a sensor of oxidative stress and a positive regulator of the human Antioxidant Response Element (ARE), regulating the expression of hundreds of gene that protect from oxidative stress^32^. NRF2 represents a complex system that can be acutely activated by either pro-oxidant compounds (i.e. ROS) or pro-inflammatory kinases. However, under chronic conditions such as those occurring in chronic diseases, a downregulation of a series of NRF2-ARE/EpRE dependent detoxification and antioxidant systems has been described^65^. The increased anion superoxide production in Nlrp3^−/−^-HFHC supports the hypothesis that the high FFAs flux to the liver leads to PPAR α-mediated fatty acid oxidation with excessive ROS formation, not counteracted by an adequate antioxidant capacity.

**Figure 8:**
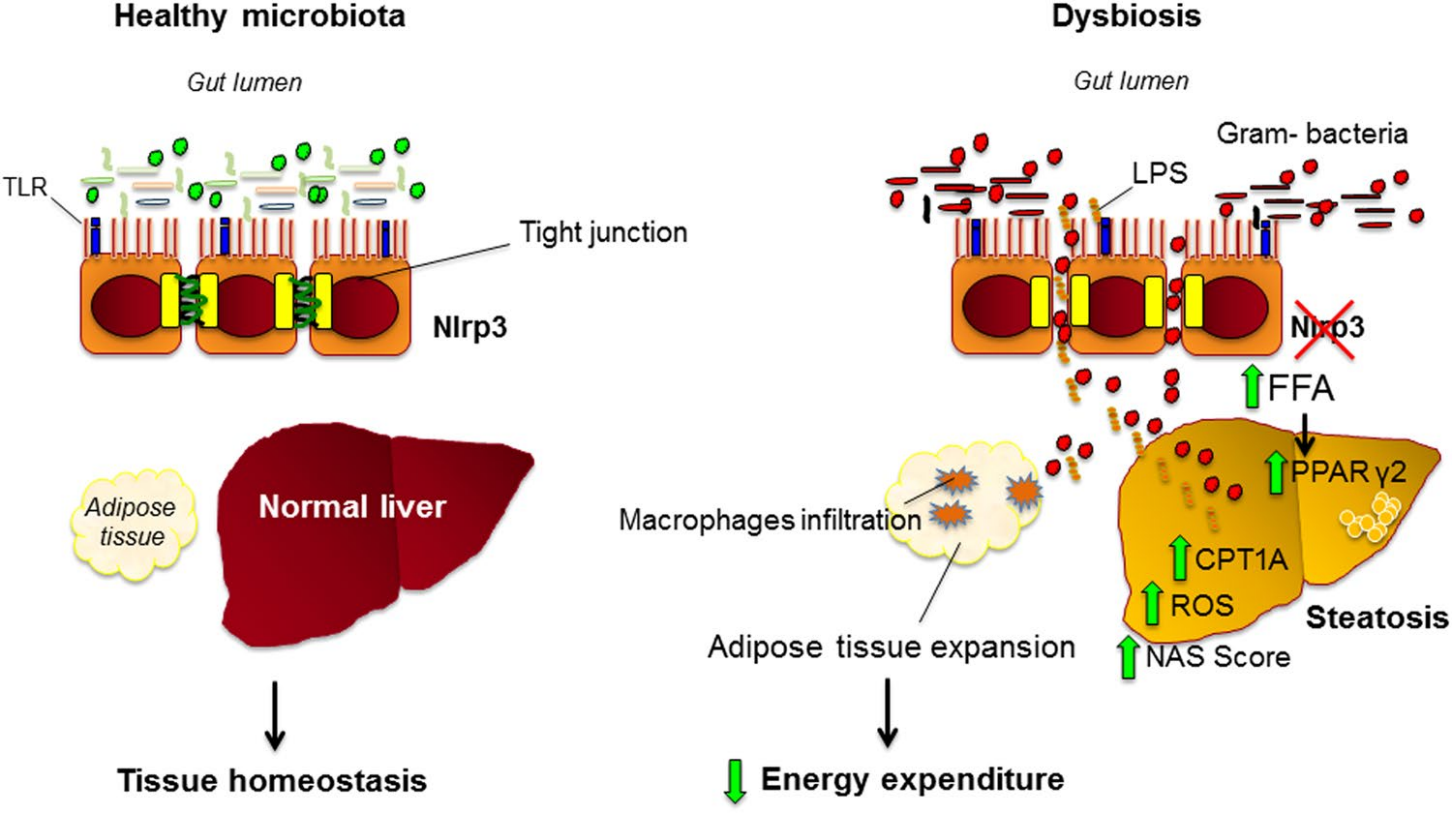
Representative scheme of the mechanisms linking lack of NLRP3 and diet in adipose tissue inflammation and progression of liver injury. NLRP3 deficiency associated with a Western-lifestyle diet drive to dysbiosis and alteration of the intestinal barrier which increase bacterial translocation. Bacterial translocation induces adipose tissue dysfunction, known to increase fatty acids efflux to the liver. Hepatic PPAR γ2 expression, responsible of fatty acids uptake, is significantly higher and is associated with increased β-oxidation, which in turn leads to ROS synthesis. In addition, circulating gram-negative bacteria trigger liver injury via specific TLRs.

To our knowledge, this is the first study that evaluates microbiota modifications in Nlrp3^−/−^-fed a Western-lifestyle diet. Taken together, our results showed that a Western-lifestyle diet induced-dysbiosis was worsened in *Nrlp3*^−/−^ mice, suggesting that inflammasome function might counteract the changes of the microbial species relative abundance. Thus, we can conclude that a combination of Western-lifestyle diet and a genetic background of defective innate immune response leads to the expansion of a pathogenetic microbiota with gram-negative bacterial translocation that drives a worsened NASH phenotype (Fig. 8).

This study introduces the basis to identify NLRP3 as a crucial target for potential manipulation of gut microbiota that may interfere with the progression of liver injury in NAFLD. In the present work, the identification of the specific cell population involved in the progression of liver injury mediated by NLRP3-inflammasome is lacking. It has been already observed^16^ that knock-in mice expressing a constitutively active NLRP3 inflammasome either in myeloid cells or hepatocytes did not influence the progression of liver injury in a MCD model. In our previous study^24^, we showed that the progression of liver fibrosis is associated with the downregulation of NLRP3 in the gut which, together with the current evidence of a strong correlation between intestinal changes (including modification of microbiota composition) and liver disease, makes the role of NLRP3 in the intestine extremely attractive as a protective factor.

## Material and Methods

### Animals

Wild-type (WT) and Nlrp3^−/−^ C57BL/6 mice were purchased respectively from Charles River Laboratories International, Inc. (Wilmington, MA, USA) and Jackson Laboratory (Bar Harbor, ME, USA). Eight weeks-old male mice were assigned to a chow or an high-fat-high-carbohydrate diet (HFHC). Mice were provided ad libitum access for 12 weeks to chow (3.1 kcal/g, 18% from fat, 24% from proteins, and 58% from carbohydrates) or HFHC (5.6 kcal/g, 58% from fat, 18% from proteins, and 24% from carbohydrates; Laboratorio Piccioni, Milan, Italy) with drinking water enriched with high-fructose corn syrup equivalent (42 g/L of carbohydrates mixed in water at a ratio of 55% fructose and 45% sucrose)^49^. A group of HFHC mice were also treated for 12 weeks with antibiotics to prevent growth of intestinal bacteria^24^. Mice were housed accordingly to their treatment and genotype. Caloric intake was expressed as calories intake/body weight ratio that was calculated from the estimated quantity of calories consumed by each mouse divided by its body weight. Animal work has been conducted according to the local committee for care and use of laboratory animals. The study protocol was approved by the Ethical Committee of the “Università Politecnica delle Marche” under the protocol named FIRB07/2011GE.

### Total energy expenditure determination

Total energy expenditure was evaluated using the method of the doubly labeled water. Briefly, mice received intraperitoneally 350 µl of water containing 318 µl of H_2_^18^O (10% enriched) and 32 µl of ^2^H_2_O (99% enriched) (Cambridge isotope, MA, USA). Blood samples from the tail vein were obtained 3 and 72 hours after injection, to evaluate enrichment after dose distribution.

### Tissue Processing and Hematoxylin and Eosin (H&E) in liver samples

Tissues were harvested and fixed in 4% paraformaldehyde overnight at 4 °C and subsequently were dehydrated and embedded in paraffin. Samples were rehydrated and stained with H&E, according to the manufacturer’s instructions (Sigma-Aldrich, St. Louis, MO, USA). Images (magnification, 20×) were acquired using a Nikon microscope (Nikon MBA75040, Florence, Italy) and scored according to Kleiner^2^.

### Hepatic and faecal triglyceride measurement

Hepatic and faecal lipid extraction has been performed according to Folch’s method^66^. Triglyceride quantification in the liver and in the stool were assessed using commercially available kits (respectively #ab65336, Abcam, Cambridge, UK and #K622-100, Biovision Inc, Milpitas, CA, USA). The absorbance has been quantified by using Sunrise spectrophotometer instrument, (Tecan Austria Gmbh. Untersbergstr, 1 A. A-5082 Grodig, Austria) at λ = 570 nm.

### Sample preparation and immunohistochemistry in adipose tissue

Epididymal fat was dissected and immediately fixed by immersion in 4% paraformaldehyde in 0.1 M phosphate buffer (PB; pH 7.4) overnight at 4 °C. After a thorough rinse in PB, specimens were dehydrated in ethanol, cleared in xylene and embedded in paraffin. Serial paraffin sections 3 μm in thickness were obtained from each specimen. After dewaxing, antigen retrieval was achieved with a pressure cooker treatment (90 °C for 20 min) by soaking sections in a sodium citrate buffer 0.01 M, pH 6.0. After a thorough rinse in phosphate buffered saline (PBS), sections were reacted with 0.3% H_2_O_2_ (in PBS; 30 min) to block endogenous peroxidase, rinsed with PBS and incubated in a 3% blocking solution (in PBS; 60 min). Then, they were incubated with the mouse monoclonal anti-MAC-2 (#CL8942AP, Cedarlane Laboratories, Paletta Court, Burlington, Ontario, Canada; dilution 1:1500), overnight. Sections were incubated in a 1:200 v/v biotinylated horse anti-mouse IgG secondary antibody solution (Vector Laboratories, Burlingame, CA, USA) in PBS, for 30 min. Histochemical reactions were performed using Vectastain ABC Kit (Vector Laboratories, Burlingame, CA, USA) and Sigma Fast 3,3′-diaminobenzidine (Sigma-Aldrich, St. Louis, MO, USA) as the substrate. Sections were finally counterstained with Hematoxylin, dehydrated and mounted in Entellan. Staining was never observed when the primary antibody was omitted. For morphometric analysis, MAC-2 immunoreactions were performed under standardized conditions for all samples. The number of MAC-2-positive crown-like structures (CLS) in the epididymal depot was evaluated on three representative sections collected every 200 µm for each animal. CLS density was calculated as CLS number/10,000 adipocytes.

### Quantitative Real-time Reverse Transcription-Polymerase Chain Reaction (qRT-PCR)

Total RNA was extracted from adipose tissue, liver or intestine using TRIzol^®^ Reagent (#15596026, Life Technologies Corporation, Woburn, MA, USA) and reverse-transcribed to complementary cDNA with the use of High Capacity cDNA Reverse Transcription Kit (#4374967, Applied Biosystems, Foster City, CA, USA). Quantitative reverse-transcription polymerase chain reaction (qRT-PCR) was performed using a Rotor-Gene 6000 instrument (Corbett Life Science Pty. Ltd., Mortlake, NSW, Australia). Relative abundance of the target genes was normalized to Cyclophilin A (PPIA) as internal control.

### Plasmatic free fatty acid composition

Plasmatic free fatty acid composition was evaluated by GC-MS (#GC7890-MS5975, Agilent technology) according to Folch’s method^66,67^. Briefly, lipid fraction was isolated using a methanol and chloroform (2:1) solution. Fatty acids were derivatized to methyl-esters with a solution of methanol-BF3 14% and dried. Samples were reconstituted in 100 µl of heptane, and 1 µl was injected in GC-MS. Single fatty acid concentration was determined using a mix of 13C labeled FFA (Alga Mix, CIL Cambridge MA, USA) as internal standard and heptadecanoic acid. Thus, saturated (palmitic 16:0 and stearic 18:0 acids) and unsaturated (palmitoleic 16:1, oleic 18:1, linoleic 18:2 and arachidonic 24:2 acids) fatty acid concentration was quantified. We then calculated the 16:0/18:1 ratio and the 16:1/16:0 ratio that are considered indexes of *de novo* lipogenesis and desaturation, respectively^63^.

### ROS staining of liver sections

Staining with dihydroethidium (#D7008, DHE, Sigma-Aldrich, St. Louis, MO, USA) was performed to determine superoxide generation in unfixed frozen liver sections. Slides were incubated for 15 minutes at 37 °C with 2 µM DHE, diluted PBS from 20 mM DHE stock solution in dimethyl sulfoxide. Slides were washed with ice-cold PBS and coverslipped. Images (magnification, 20×) were acquired using a Nikon fluorescent microscope (Nikon MBA75040, Florence, Italy), Rodamin filter and the fluorescence intensity was quantified^68^.

### Assessment of bacterial translocation

Bacterial translocation was determined by evaluation of turbidity of mesenteric lymph nodes culture. Mesenteric lymph nodes were aseptically removed from mice abdomen, equal quantities were suspended in 2 ml of sterilized Lysogeny Broth (#12780052, LB, Thermo Fisher Scientific, IL, USA) and kept for 6 hours at 37 °C with gently agitation. Differences in bacterial growth were determined spectrophotometrically (OD, optical density 600 nm) as previously reported^24^.

### Western blotting

SDS-PAGE electrophoresis and Western blotting were performed as previously described^24^. Nitrocellulose sheets where incubated with 1:200 anti-Zonulin-1 (#ab59720, Abcam, Cambridge, UK) and 1:200 anti-Occludin (sc-133256, Santa Cruz, Dallas, Texas, USA). Blots were visualized using the ECL detection system according to manufacturer’s instructions (#32132, Thermo Fisher Scientific, IL, USA) and quantified by scanning video densitometry using the ChemiDoc-It HR 410 Imaging system (UVP LLC, Upland, CA, USA). Blots were re-probed with anti-β Actin mouse antibody (#A5316, Sigma Aldrich, St. Louis, MO, USA) to demonstrate equal loading.

### Gut microbiome metagenomic analysis

Caecal content was collected at the end of treatment and immediately stored at −80 °C. Each sample was subjected to DNA extraction according to QIAamp Fast Stool Kit protocol (#51604, QIAGEN, Hilden, Germany). The extracted DNA was quantified on a Qubit 2.0 Fluorometer (Thermo Fisher Scientific, IL, USA) using the Qubit ds DNA High Sensitivity Assay Kit (#Q32851, Thermo Fisher Scientific, IL, USA). DNA integrity was confirmed on 0.8% agarose gel (Sigma Aldrich, St. Louis, MO, USA). Amplification of the V4 region of 16S rDNA was based on a protocol published by Caporaso and co-workers^69^. Two separate V4 rRNA gene amplification reactions were performed, pooled together, cleaned up using AMPure XP magnetic beads (#A63881, Beckman Coulter, Brea, CA, USA) and quantified with Qubit HS assay (#32855, Thermo Fisher Scientific, IL, USA). Genomic DNA samples extracted from the caecal content of the antibiotic treated animals were subjected to 2 consecutive rounds of DNA amplification due to the scarcity of bacterial DNA as a consequence of the lowered bacterial load in the samples. Libraries were constructed according the 16S Metagenomic Sequencing Library Preparation protocol (Illumina, San Diego, CA, USA). Normalized sample libraries were pooled and loaded onto the Illumina MiSeq cartridge according to the manufacturer’s instructions using MiSeq Reagent Kit v3 (2 × 300 bp Paired-End Reads, 15 Gb output). FastQ files were generated at the end of the run to perform the quality control. The quality of the run was checked internally using PhiX Control and then each pair-end sequence was assigned to its sample using the multiplexing index. Therefore, the paired-reads with a minimum overlap of eight bases were merged using a specific QIIME 1.9.1 scripts. OTUs generation was done using a QIIME pipeline based on USEARCH’s OTUs clustering recommendations (http://www.drive5.com/usearch/manual/otu_clustering.html). Reads were clustered at 97% identity using UCLUST to produce OTUs^70^. Taxonomy assignment of resulting OTUs was performed using the Greengenes 13_8 database^71^. Rarefaction, to a subsampling depth of 14,123 reads/sample, was performed on all samples to standardize the sequencing depth. Richness and α-diversity were quantified as observed OTU counts and Shannon diversity index; the statistical significance of differences in microbial community composition index between sample categories was determined by non parametric Monte Carlo permuted t-test two-sided (999 permutation) and Benjamini-Hochberg false discovery rate test correction.For β-diversity, Unweighted UniFrac distance matrix among all samples was calculated as input to principle coordinates analysis (PCoA). Statistical significance of the sample groupings was tested with non parametric ANOSIM (999 permutation). To determine if statistically significant differences occurred in microbial populations between the groups, non parametric t-test analysis was completed; p values were corrected for multiple inference using Benjamini-Hochberg false discovery rate procedure and a adjusted alpha cutoff value of 0.05. Hierarchical cluster analysis was obtained with Euclidian distance and Ward method.

## Electronic supplementary material

Supplementary information
