# Author Correction: Lack of NLRP3-inflammasome leads to gut-liver axis derangement, gut dysbiosis and a worsened phenotype in a mouse model of NAFLD

**DOI:** 10.1038/s41598-017-17187-3

**Published:** 2017-12-11

**Authors:** Irene Pierantonelli, Chiara Rychlicki, Laura Agostinelli, Debora Maria Giordano, Melania Gaggini, Cristina Fraumene, Chiara Saponaro, Valeria Manghina, Loris Sartini, Eleonora Mingarelli, Claudio Pinto, Emma Buzzigoli, Luciano Trozzi, Antonio Giordano, Marco Marzioni, Samuele De Minicis, Sergio Uzzau, Saverio Cinti, Amalia Gastaldelli, Gianluca Svegliati-Baroni

**Affiliations:** 1 Department of Gastroenterology, Università Politecnica delle Marche, Ancona, Italy; 2 Cardiometabolic Risk Lab, Institute of Clinical Physiology, National Council of Research (CNR), Pisa, Italy; 3 Porto Conte Ricerche, Parco Scientifico e Tecnologico della Sardegna, Alghero, Italy; 4 Department of Biomedical Sciences, Università di Sassari, Sassari, Italy; 5 Department of Experimental and Clinical Medicine, Università Politecnica delle Marche, Ancona, Italy; 6 Obesity Center, Università Politecnica delle Marche, Ancona, Italy

*Scientific Reports* **7**:12200; doi:10.1038/s41598-017-11744-6; Article published online 22 September 2017

The original version of this Article contained errors in the spelling of the authors Irene Pierantonelli, Chiara Rychlicki, Laura Agostinelli, Debora Maria Giordano, Melania Gaggini, Cristina Fraumene, Chiara Saponaro, Valeria Manghina, Loris Sartini, Eleonora Mingarelli, Claudio Pinto, Emma Buzzigoli, Luciano Trozzi, Antonio Giordano, Marco Marzioni, Samuele De Minicis, Sergio Uzzau, Saverio Cinti, Amalia Gastaldelli & Gianluca Svegliati-Baroni which were incorrectly given as Pierantonelli Irene, Rychlicki Chiara, Agostinelli Laura, Giordano Debora Maria, Gaggini Melania, Fraumene Cristina, Saponaro Chiara, Manghina Valeria, Sartini Loris, Mingarelli Eleonora, Pinto Claudio, Buzzigoli Emma, Trozzi Luciano, Giordano Antonio, Marzioni Marco, De Minicis Samuele, Uzzau Sergio, Cinti Saverio, Amalia Gastalderi & Svegliati-Baroni Gianluca.

These errors have now been corrected in the HTML and PDF versions of the Article, and in the accompanying Supplementary Information document.

