# Supplementary material for: Lack of NLRP3-inflammasome leads to gut-liver axis derangement, gut dysbiosis and a worsened phenotype in a mouse model of NAFLD
Source: Sci Rep. 2017 Sep 22;7:12200. doi: 10.1038/s41598-017-11744-6 (PMC5610266; doi:10.1038/s41598-017-11744-6)
Supplement: Supplementary file 1 — Supplementary information [file 41598_2017_11744_MOESM1_ESM.doc]

**LACK OF NLRP3-INFLAMMASOME LEADS TO GUT-LIVER AXIS DERANGEMENT, GUT DYSBIOSIS AND A WORSENED PHENOTYPE IN A MOUSE MODEL OF NAFLD.**

Pierantonelli Irene^1^, Rychlicki Chiara^1^, Agostinelli Laura^1^, Giordano Debora Maria^1^, Gaggini Melania^2^, Fraumene Cristina^3^, Saponaro Chiara^2^, Manghina Valeria^3,4^ Sartini Loris^5^, Mingarelli Eleonora^1^, Pinto Claudio^1^, Buzzigoli Emma^2^, Trozzi Luciano^1^, Giordano Antonio^5^, Marzioni Marco^1^, De Minicis Samuele^1^, Uzzau Sergio^3,4^, Cinti Saverio^5,6^, Gastaldelli Amalia^2^, Svegliati-Baroni Gianluca^1,6*^.

^1^Department of Gastroenterology, Università Politecnica delle Marche, Ancona, Italy.

^2^Cardiometabolic Risk Lab, Institute of Clinical Physiology, National Council of Research (CNR), Pisa , Italy.

^3^Porto Conte Ricerche, Parco Scientifico e Tecnologico della Sardegna, Alghero, Italy.

^4^Department of Biomedical Sciences, Università di Sassari, Sassari, Italy.

^5^Department of Experimental and Clinical Medicine, Università Politecnica delle Marche, Ancona, Italy.

^6^ Obesity Center, Università Politecnica delle Marche, Ancona, Italy.

**Correspondence**

Prof. Gianluca Svegliati-Baroni, Department of Gastroenterology, Università Politecnica delle Marche, Via Tronto 10, 60126 Ancona, Italy, phone: +39 0712206043, fax: +39 0712206044, e-mail: [gsvegliati@gmail.com](mailto:gsvegliati@gmail.com)

**
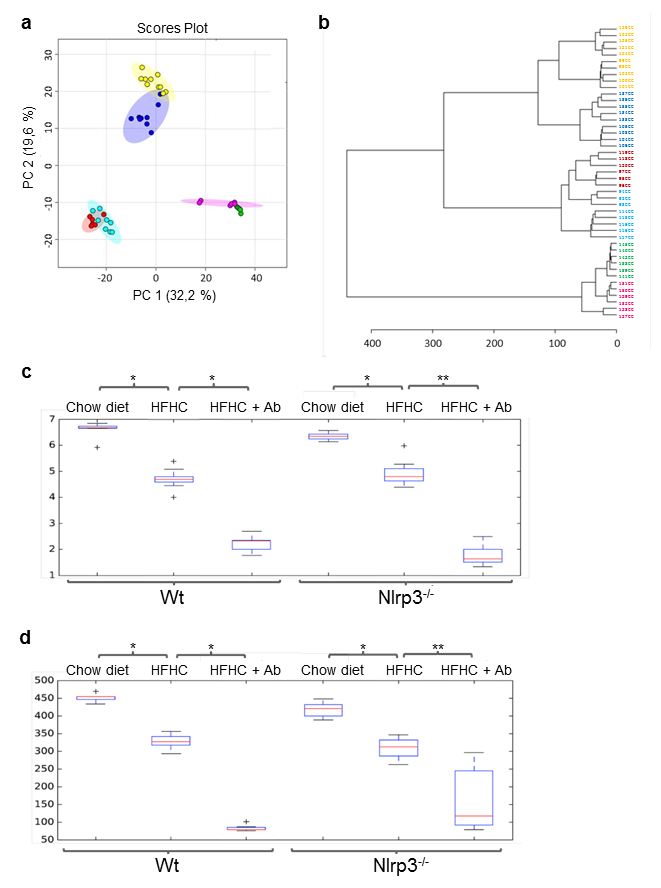
**

**SUPPLEMENTARY FIGURE S1. α-diversity, richness and β-diversity analysis of the microbiota community.**  PCA groups representation (ANOSIM p-value = 0.001) (a). Unsupervised clustering analysis (yellow: Wt-HFHC; dark blue: Nlrp3^-/-^-HFHC; red: Nlrp3^-/-^-Chow diet; light blue: Wt-Chow diet; green: Nlrp3^-/-^-HFHC + Ab; magenta: Wt-HFHC + Ab) (b). Shannon index (c) and observed OTUs number (d) boxplots. * p<0.05; ** p<0.01.

**
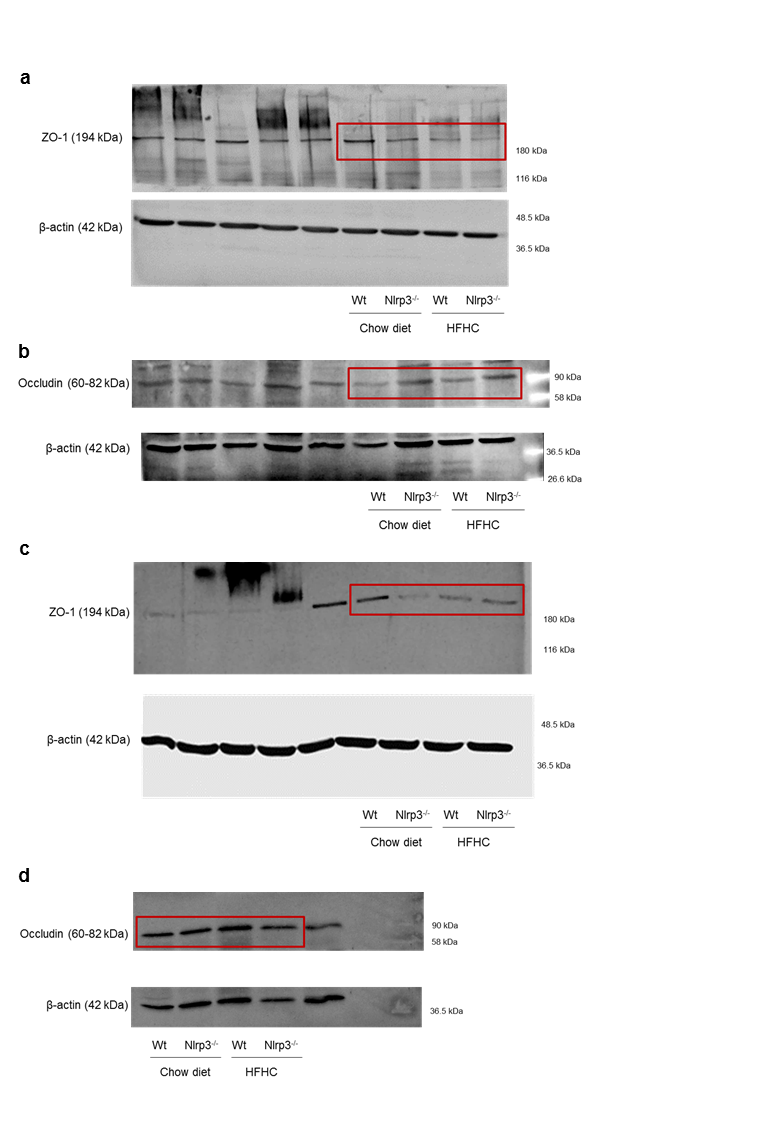
**

**SUPPLEMENTARY FIGURE S2. Uncropped Western blot images.**  (a-b) This figure represents full-lenght Western blot images of original Fig 5b; (c-d) this figure represents full-lenght Western blot images of original Fig 5c.
